# Transdiagnostic clinical correlates for behaviors of concern in pediatric neurodevelopmental disorders: a retrospective chart review and machine learning analysis

**DOI:** 10.3389/fpsyt.2026.1739597

**Published:** 2026-08-12

**Authors:** Myka Estes, Margaret Ryan, Sara Early, Kimberly Amador, Noah Bloom, Amani Ahmed, Anza Momin, Tina R. Ram, Nils D. Forkert, Sarah J. MacEachern

**Affiliations:** 1 Department of Pediatrics, Cumming School of Medicine, University of Calgary, Calgary, AB, Canada; 2 Alberta Children’s Hospital Research Institute, Cumming School of Medicine, University of Calgary, Calgary, AB, Canada; 3 Department of Radiology, University of Calgary, Calgary, AB, Canada; 4 Hotchkiss Brain Institute, University of Calgary, Calgary, AB, Canada; 5 Biomedical Engineering Graduate Program, University of Calgary, Calgary, AB, Canada

**Keywords:** behaviors of concern, chart review, clinical outcomes, machine learning, medical complexity, neurodevelopmental disorders, pediatrics, transdiagnostic

## Abstract

**Introduction:**

Behaviors of concern, such as aggression and self-injury, are common in children with neurodevelopmental disorders and contribute substantially to caregiver stress and health system burden. However, most evidence on behaviors is drawn from diagnostically homogeneous samples, limiting relevance to real-world presentations encountered in tertiary care. Our aim was to characterize the diagnostic and behavioral complexity of children referred to tertiary clinics and use machine learning to identify transdiagnostic features associated with behaviors across diverse neurodevelopmental presentations.

**Method:**

This retrospective chart review and analysis examined all children aged 2 to 17 years undergoing first-time assessment at a tertiary developmental pediatrics clinic between May 2022-2023. Unstructured physician notes and supporting documentation were transformed into structured data and supervised machine learning models identified factors associated with behaviors of concern.

**Results:**

Among 600 children, 83% exhibited at least one behavior of concern (mean, 3.15 [SD 2.98]). Most had multiple neurodevelopmental diagnoses (73%) and frequent co-occurrence of physical (80%) and mental health conditions (21%). Emotional dysregulation and non-cooperation were the most common behaviors, while aggression and self-injury were frequently moderate to severe. Machine learning models identified sleep difficulties, speech and language delay, restricted and repetitive behaviors (B symptoms) of autism, gastrointestinal issues, and service use as top-ranked associated factors across multiple behavioral types. Factors associated with any behaviors included ADHD, sensory impairments, and genetic contributors to autism.

**Discussion:**

Transdiagnostic factors highlight key domains often under-assessed in routine care. These findings support integrated assessment models that address modifiable clinical correlates for behaviors of concern across diagnostic boundaries to improve outcomes for high-need neurodevelopmental populations.

## 1 Introduction

Behaviors of concern (BoC), such as aggression and self-injury, affect up to 80% of children with neurodevelopmental disorders (NDDs) and represent some of the most disruptive and treatment-resistant challenges in this population (1–3). These behaviors often drive referrals to tertiary care, strain families and school systems, and necessitate intensive, costly services (4–9). The burden of care falls heavily on caregivers and public systems, contributing to diminished quality of life and markedly increased healthcare expenditures, estimated to be 2 to 5 times higher for children with severe BoC (10–13).

Children seen in tertiary developmental clinics rarely present with a single NDD. Instead, they often carry multiple diagnoses, including autism, attention-deficit/hyperactivity disorder (ADHD), intellectual disability, communication disorders, and motor delays. This diagnostic complexity is further compounded by frequent co-occurring physical health conditions (14–16) and mental health concerns (17–19). Recent genetic and neurobiological evidence supports this transdiagnostic reality, revealing shared etiological pathways across traditionally distinct NDD categories (20).

Despite these overlapping clinical and biological features, most research on BoC continues to focus on single-disorder cohorts, particularly autism. Even robust associations, such as links between sleep or gastrointestinal (GI) problems and BoC, have largely been established within diagnostically narrow populations (21, 22). This creates a profound mismatch between research and practice: clinicians must support children with layered, intersecting challenges, but are left drawing inferences from siloed evidence that excludes the very complexity that defines real-world care (20, 23).

To address this disconnect between research evidence and clinical reality, we conducted a retrospective chart review of children presenting for first-time assessment in tertiary developmental pediatrics clinics over a one-year period. Using structured data abstraction, we captured neurodevelopmental, physical, and mental health diagnoses, BoC, and key contextual factors, including sleep problems, family structure, and clinical and social support engagement. We then used supervised machine learning models to examine whether consistent, multivariate features associated with BoC could be identified across this diagnostically complex population. Our primary research question was: Can we identify transdiagnostic features that predict the presence of BoC in children undergoing tertiary assessment? In addressing this question, we aimed to bridge the gap between narrowly framed research and the multidimensional populations encountered in everyday clinical practice.

## 2 Method

### 2.1 Design

We conducted a retrospective chart review of children aged 2 to 17 years who underwent first-time diagnostic assessments in subspecialty developmental pediatrics clinics at Alberta Children’s Hospital, between May 2022 and May 2023. Children were eligible if they received an NDD diagnosis based on a comprehensive clinical assessment and were seen by a developmental pediatrician or pediatric neurologist (**Supplementary Materials**). The use of standardized instruments to support diagnoses are not reported as not every patient underwent such assessment and, if completed, the results were not always available. NDDs are defined as conditions that affect the developing brain (24) and therefore in addition to DSM-5 classifications, conditions such as cerebral palsy are defined as NDDs in this study. This pragmatic sample reflects the diagnostic and clinical heterogeneity typical of tertiary developmental pediatrics, where children commonly present with multiple, overlapping neurodevelopmental, medical, and behavioral concerns. Ethics and operational approvals were obtained from CHREB at the University of Calgary (REB23-0405) and AHS Data Disclosure Agreement and Administrative Approval.

### 2.2 Data abstraction from clinician notes

Five raters manually abstracted information from electronic health records (EHRs). Diagnostic conclusions were gathered from free-text clinician notes. Standardized assessments were conducted for some patients and included in the diagnostic formulation by the physician where appropriate, but were not reported in this study due to the inconsistency between patients and the results of certain standardized assessments not always being in alignment with the final diagnostic conclusion of the assessing physician [*e.g.*, a child scoring in the range for autism on the Autism Diagnostic Observation Schedule [ADOS-2] due to co-occurring conditions (25)]. While all raters were trained on data extraction standards and provided a manual of operations outlining collection procedures, we recognize the inherent limitations in the secondary use of EHRs for research purposes (26, 27). Clinician notes reflect both the caregiver’s report, and the clinician’s interpretation of the child’s state and therefore biases in reporting may be present. Furthermore, while our raters were trained on data extraction standards, further biases in our interpretation of the clinician’s notes may be present. Nonetheless, EHRs offer rich data and the acknowledgement of these potential limitations is important to contextualize the analyses and conclusions.

### 2.3 Data abstraction for behaviors of concern

Seventy-one behavioral topographies across seven behavioral categories were abstracted from EHRs. These behaviors were derived from established measures—Behaviour Problems Inventory (BPI-01) (28), the Problem Behaviour Checklist (PBCL) (29), as well as clinical expertise. Behaviors were identified through review of unstructured physician notes, and each documented behavior was assessed for its severity. Severity ratings were assigned as mild, moderate, or severe according to the following operational criteria:

- Mild: Behavior occurs but does not cause significant harm (*e.g.*, temporary reddening of the skin, very light bruising). Alternatively, the behavior produces signs or symptoms that are easily tolerated, transient, and do not require therapy or medical evaluation. No disruption to daily activities occurs.
- Moderate: Behavior may cause moderate harm (*e.g.*, moderate bruising, scratching through the skin, repeated picking of scabs) or introduces a low level of inconvenience or concern. These events may interfere with daily activities but are generally manageable with simple therapeutic measures.
- Severe: Behavior causes moderate to severe injury (*e.g.*, biting through the skin, eye gouging, bone fractures) and typically requires medical intervention. Alternatively, the behavior substantially disrupts daily activities, often necessitating systemic therapy or other intensive treatment, and is usually incapacitating.

In addition to rating the severity of each documented behavior individually, we assigned an overall BoC profile severity rating for each child. This overall rating reflected the highest severity level observed across all of the child’s behaviors. For example, a child exhibiting multiple mild behaviors and one severe behavior would be classified as having a severe overall profile. This approach aligns with clinical practice, where treatment planning and resource allocation are typically driven by the most concerning behavior present, rather than the average severity across all behaviors.

### 2.4 Machine learning dataset

For each category of BoC (*e.g.*, aggression, self-injury), patients were divided into two cohorts based on whether they exhibit the specific behavior or not. 147 features were considered for each BoC classification task, encompassing demographics, neurodevelopmental and psychiatric conditions, medical history, and support services. To appropriately handle any missing data, two different methods were used for data imputation depending on the input feature type. For categorical features, a unique label was created in place of the missing information. For continuous features, the missing data were imputed with the mean value of the feature of interest. These variables were then entered into an established machine learning pipeline to develop a separate predictive model for each BoC with the identification of key factors associated with each outcome.

### 2.5 Machine learning analysis

To identify which clinical features distinguish children with and without specific BoC, we conducted eight distinct machine learning experiments—one for each behavior of interest—using an in-house developed pipeline (30, 31). This pipeline consists of two main stages: feature ranking and selection, which identified key predictive factors associated with the specific BoC, followed by binary classification, which predicted presence or absence of each specific BoC within the patient cohort based on the selected features.

In the feature ranking and selection stage, the ReliefF algorithm was employed to rank the importance of features separately for each binary classification task. This algorithm was chosen due to its effectiveness in handling high-dimensional data and its ability to identify relevant features while mitigating the impact of noise (32, 33). Briefly described, ReliefF iteratively adjusts feature weights based on the differences between nearest neighbor instances from the same and opposite classes, making it well-suited for datasets with a large number of features. ReliefF is particularly well-suited for datasets containing hierarchically nested features (*e.g.*, a general diagnostic category and its specific subtypes). Unlike correlation-based feature selection methods that may treat such nested variables as redundant, ReliefF evaluates each feature independently based on its ability to distinguish between nearest neighbors of the same versus different classes. For classification, a logistic model tree (LMT) was utilized. This hybrid machine learning approach integrates decision tree structures with logistic regression, balancing interpretability with strong predictive performance (34). To optimize the final model for each task, we selected the subset of features that yielded the highest classification accuracy for that specific task by iteratively removing the least important feature and then re-training and evaluating the classifier with the reduced feature set.

To ensure robustness and generalizability, we implemented a 10-fold cross-validation strategy. Therefore, the dataset was divided into 10 equal subsets, with one-fold reserved for testing while the remaining nine were used for training. This process was repeated across all folds to ensure comprehensive model evaluation. To address class imbalance between children with and without each specific BoC, we employed random under-sampling of the majority class to match the minority class size, repeated across 30 iterations with different random samples to ensure stability. Final performance metrics were obtained by averaging results across the 10 folds. Practically, the feature ranking and selection was nested within cross-validation to prevent data leakage and ensure an unbiased model evaluation.

### 2.6 Machine learning domain groupings

**Figure 1:**
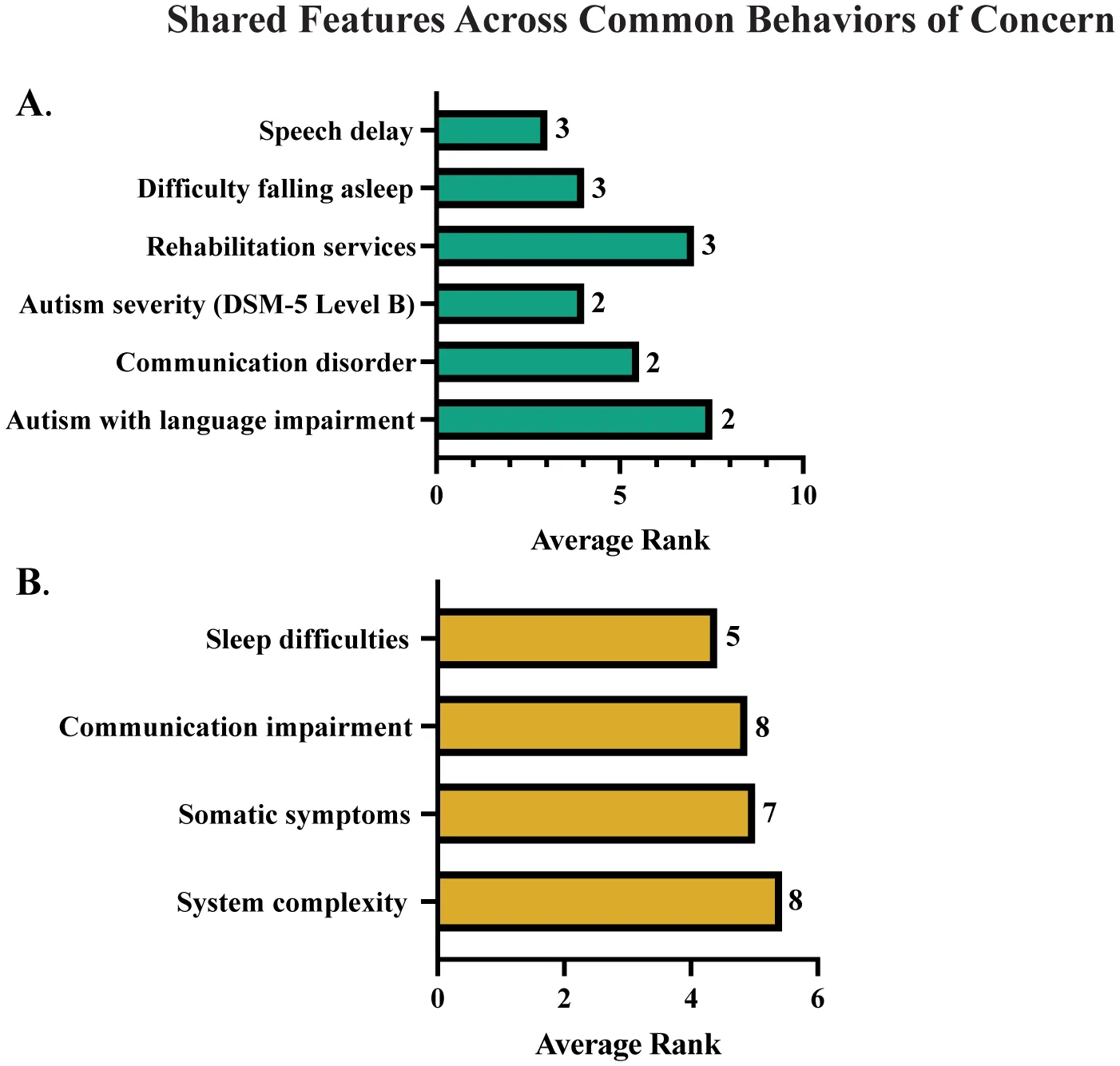
Shared features across common behaviors of concern. **(A)** Individual features that ranked among the top 10 in at least two of the four most common BoC types. Bar length indicates average rank across behaviors (shorter bars = higher importance). Annotations show number of BoC types in which each feature appeared. **(B)** Domain-level summary showing average ranking and frequency of appearance for grouped clinical features. Bars show average rank for all features within each domain when appearing in top 10 (shorter = more predictive); annotations indicate total number of top-10 appearances across all BoC types.

To examine broader clinical patterns in features associated with BoC, individual features were grouped into four clinically relevant domains: Somatic Symptoms, System Complexity, Communication Impairment, and Sleep Difficulties. These domains were determined based on clinical relevance and conceptual similarity of the model features and were used for summarizing model results across behavior types. Features assigned to the Somatic Symptoms domain captured the presence of physical health problems outside the context of sleep. These included features such as chronic pain, GI disturbances, motor abnormalities, and sensory sensitivities. Features assigned to the System Complexity domain reflected the extent of service involvement and the structural burden of care. This included enrollment in disability-related supports (*e.g.*, the Disability Tax Credit, Family Support for Children with Disabilities), engagement with pediatric rehabilitation services, and the total number of formal supports active at the time of assessment. While these variables do not directly quantify clinical severity, they serve as proxies for healthcare utilization, chronicity of need, and the administrative complexity often accompanying high co-occurrence rates and functional impairment. The Communication Impairment domain encompassed features related to expressive, receptive, and pragmatic communication difficulties. Features in this domain included expressive language delay, receptive language delay, speech production impairments, and deficits in nonverbal communication. The Sleep Difficulties domain grouped features describing disruptions to sleep initiation, maintenance, and quality, including insomnia symptoms, prolonged sleep latency, frequent night wakings, and irregular sleep patterns. Each feature was assigned exclusively to a single domain based on its primary clinical association. For domain-level aggregation presented in **Figure 1B**, two summary metrics were calculated:

1. Average Rank: For each domain, we calculated the mean rank across all individual features belonging to that domain, considering only appearances where the feature ranked among the top 10 most important features for a given BoC.
2. Top-10 Appearance Count: We computed the total number of times any feature within a domain appeared among the top 10 features across all BoC.

## 3 Results

### 3.1 Sample characteristics

We conducted a retrospective chart review of children and youth who underwent first-time diagnostic assessment between May 2022 and May 2023 across seven subspecialty clinics within Alberta Children’s Hospital’s tertiary Child Development Services. These clinics provide comprehensive diagnostic evaluation for children with complex neurodevelopmental, medical, genetic, and behavioral presentations referred when diagnostic uncertainty or clinical complexity exceeds community capacity. Only first-time assessments were included to standardize baseline diagnostic profiles. A total of 636 medical charts were reviewed, of which 600 met eligibility criteria. Children had a median (IQR) age of 5.7 (3.8-9.8) years, and 71% (n = 424) were male. Nearly all children (99%) were cisgender, and 89% lived in households with at least one biological parent. Neighborhood socioeconomic status was assessed using the MSDI, which links patients’ residential postal codes to area-based deprivation scores derived from Canadian census data. Based on MSDI ratings, 29% of children lived in neighborhoods characterized by material deprivation but social privilege (**Table 1****).**

**Table 1:** Demographics.

Child demographics.	n = 600 (%)
Age, median (IQR)	5.7 (3.8-9.8)
Sex	
Male	424 (71)
Female	176 (29)
Gender	
Cisgender	596 (99)
Transgender	7 (1)
Neurodevelopmental disorder (NDD) diagnosis	
Autism	389 (65)
Communication disorder	264 (44)
Intellectual disability	253 (42)
ADHD	215 (36)
Motor disorder	58 (10)
Specific learning disorder	49 (8)
ND-PAE	15 (2)
Cerebral Palsy	6 (1)
Number of NDD diagnoses	
1	164 (27)
2 or more	436 (73)
Number of co-occurring NDDs, median (IQR)	2 (1-3)
Physical health conditions^1^	
Sleep problem	274 (46)
Gastrointestinal	215 (36)
Vision	122 (20)
Allergies	85 (14)
Congenital/genetic disorder	82 (14)
Sleep disorder	73 (12)
Asthma	48 (8)
Seizures	46 (8)
Dermatological	40 (7)
Audiologic	39 (7)
Dental	38 (6)
Hematological/Oncology	29 (5)
Headache disorders	28 (5)
Cardiovascular	25 (4)
MSK	20 (3)
GU	18 (3)
Endocrine	13 (2)
ENT	11 (2)
Renal	9 (2)
Obesity	7 (1)
Autoimmune	≤5 (1)
Chronic infection	≤5 (1)
Respiratory	≤5 (1)
Transplant	≤5 (1)
Number of physical gealth conditions	
1	154 (26)
2 or more	324 (54)
Number of co-occurring Physical, median (IQR)	2 (1-3)
Mental health diagnoses^2^	
Anxiety	95 (16)
ODD	18 (3)
Eating disorder	16 (3)
Depression	13 (2)
Trauma/PTSD	7 (1)
OCD	≤5 (1)
Pica	≤5 (1)
Attachment	≤5 (1)
Mutism	≤5 (1)
SAD	≤5 (1)
DMDD	≤5 (1)
Trichotillomania	≤5 (1)
Adjustment disorder	≤5 (1)
Conduct disorder	≤5 (1)
Schizophrenia	≤5 (1)
Phobia	≤5 (1)
Gender dysphoria	≤5 (1)
Number of mental health diagnoses	
1	87 (15)
2 or more	38 (6)
Number of co-occurring Mental, mean (SD)	0.3 (0.7)

Family demographics	
Number of siblings	
1	258 (43)
2	134 (22)
3 or more	87 (14)
Parental status	
Both biological parents involved	460 (76)
One biological parent involved	81 (13)
Neither biological parent involved	51 (9)

Household characteristics	
Material and social deprivation index (MSDI)^3^	
Materially and socially privileged	118 (20)
Average material and social deprivation	140 (23)
Materially privileged but socially deprived	96 (16)
Socially privileged but materially deprived	173 (29)
Materially and socially deprived	50 (8)

^1^Missing data for 45 participants.

^2^Missing data for 11 participants.

^3^Missing data for 26 participants.

Missing data suppressed due to small (≤5) cell size.

ADHD, attention-deficit/hyperactivity disorder; DMDD, disruptive mood dysregulation disorder; ENT, ear, nose, and throat; GU, genitourinary; IQR, interquartile range; MSDI, Material and Social Deprivation Index; MSK, musculoskeletal; ND-PAE, neurodevelopmental disorder associated with prenatal alcohol exposure; NDD, neurodevelopmental disorder; OCD, obsessive-compulsive disorder; ODD, oppositional defiant disorder; PTSD, posttraumatic stress disorder; SAD, separation anxiety disorder; SD, standard deviation.

### 3.2 Diagnostic complexity

The cohort exhibited substantial diagnostic heterogeneity. Nearly three-quarters of children (73%; 436 of 600) had two or more NDD diagnoses, with 45% having two, 20% having three, and 6% having four or more. The median number of NDD diagnoses per child was 2 (IQR, 1–3), and the mean was 2.1 (SD, 1). Among children with multiple NDDs, the most common diagnostic dyads were autism co-occurring with either ADHD or intellectual disability. For those with three or more diagnoses, the most frequent triplet included autism, ADHD, and intellectual disability. These diagnostic clusters were often accompanied by additional communication or motor disorders (**Table 1**; **Supplementary Tables 1, 2**).

### 3.3 Physical and mental health co-occurrence

Co-occurring physical health conditions were documented in 478 children (80%). The number of distinct physical diagnoses per child ranged from 0 to 15 (median, 2; IQR, 1–3). Sleep disturbances were the most frequently reported physical health issue, affecting 274 children (46%), followed by GI problems (215 children, 36%), vision problems (20%), and allergies (14%). Among those with sleep difficulties, 64% had trouble falling asleep, 44% had difficulty staying asleep, and 39% were taking sleep medications. Diagnosed sleep disorders were recorded in a smaller subset (73 children, 12%), suggesting underdiagnosis relative to caregiver-reported disturbances. Other frequently reported conditions included genetic syndromes (14%), asthma (8%), seizure disorders (8%), and dermatologic, auditory, or dental concerns (6-7%; **Table 1**; **Supplementary Table 3**).

Mental health conditions were identified in 125 children (21%). Most children with a mental health diagnosis had only one mental health condition (15%), though a small number had two (6%) or more diagnoses. The most frequently documented condition was anxiety, affecting 95 children (16%), followed by oppositional defiant disorder (3%), eating and feeding disorders (3%), and depression (2%). Less common diagnoses included post-traumatic stress disorder, obsessive-compulsive disorder, pica, and attachment difficulties, each affecting less than 1.2% of the sample (**Table 1**).

### 3.4 Behaviors of concern

**Figure 2:**
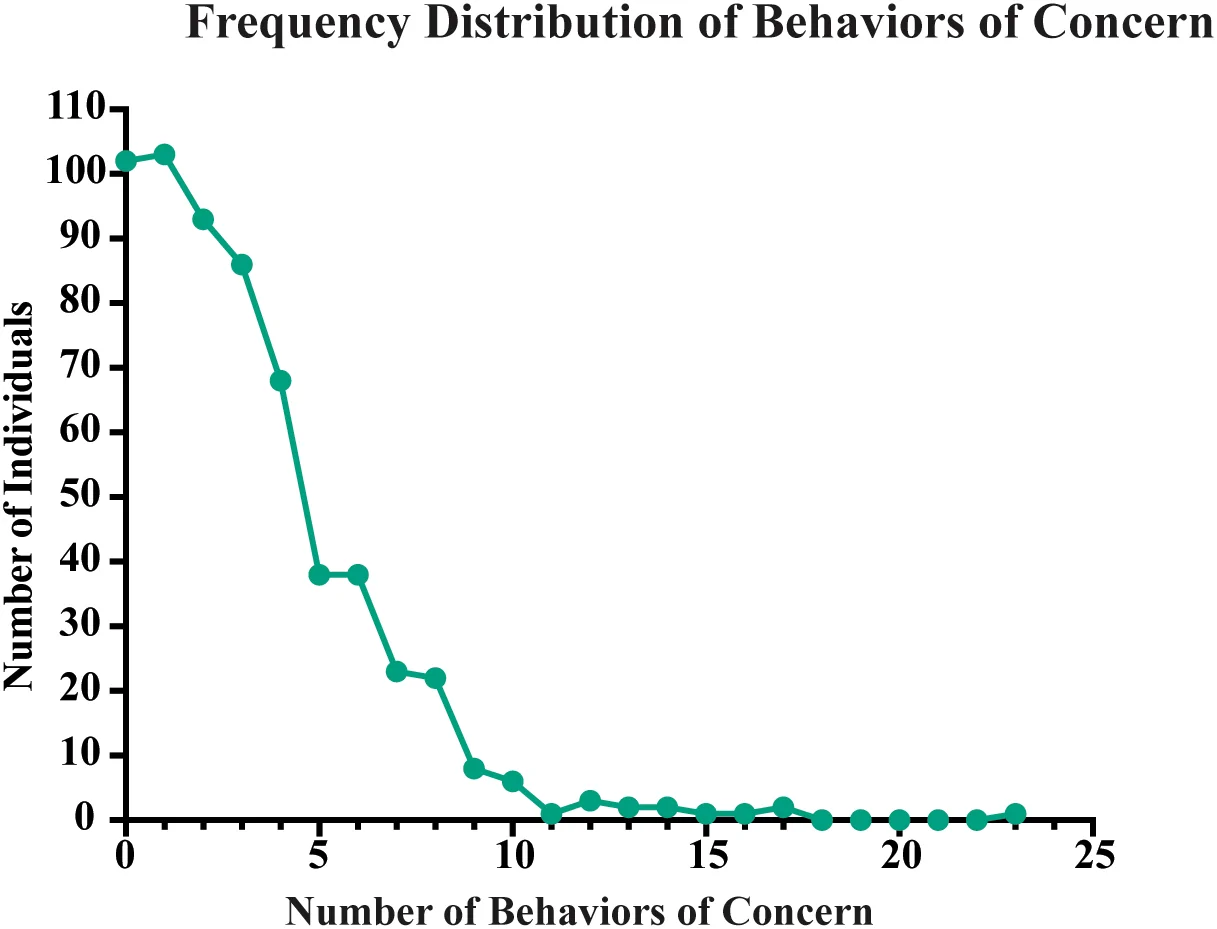
Frequency distribution of behaviors of concern. BoC are highly prevalent and multifaceted in children with NDDs in this real-world clinical sample. Distribution of the number of distinct BoC observed per child (N = 600). Most children presented with multiple behaviors, with a mean of 3.15 behaviors (SD = 2.98). Over 80% of the sample had at least one documented BoC.

BoC were present in 498 children (83%), with a median of 3 (IQR, 1-4) behaviors per child in the total population. Most children exhibited either moderate (52%) or severe (39%) overall BoC profiles, while only 9% were rated as having a mild presentation. Overall profile severity was determined by the highest severity level of any documented behavior; thus, children classified as having severe profiles may have exhibited multiple mild or moderate behaviors but had at least one behavior meeting severe criteria. This classification approach reflects the clinical reality that treatment intensity is typically guided by the most concerning behavior present. **Figure 2** shows the distribution of BoC counts across the cohort.

Detailed analysis of behavior frequency distribution revealed that of those with BoC, 36% exhibited 2-3, while 43% showed 4 or more BoC. The right-skewed distribution suggests that while most children present with multiple behaviors, a distinct subset (approximately 22%) presents with highly complex behavioral profiles involving 6 or more concurrent BoC. This pattern highlights the substantial behavioral complexity encountered in tertiary care settings.

**Figure 3:**
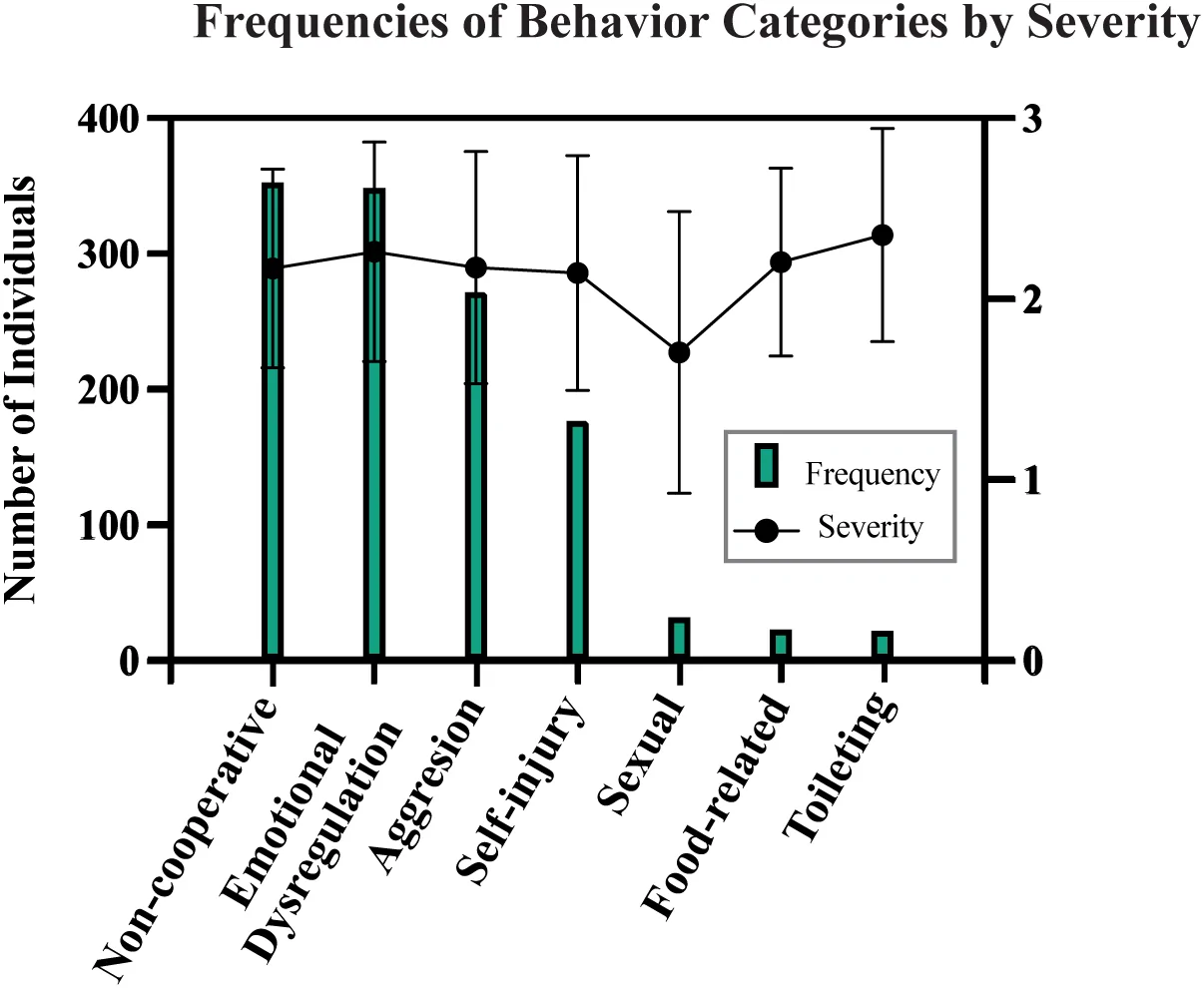
Frequencies of behavior categories by severity. Proportion of children with each category of BoC, stratified by severity (mild, moderate, severe). Emotional dysregulation and non-cooperative behaviors were the most common (70% each), followed by aggression (55%) and self-injury (35%). Notably, over half of aggressive and self-injurious behaviors were rated as moderate or severe.

The most common BoC categories were emotional dysregulation and non-cooperation, each affecting 70% of children with BoC. These were followed by aggression (55%) and self-injury (35%). Less common, albeit clinically relevant, behaviors included inappropriate sexual behaviors (6%), toileting issues (4%), and food-related concerns (4%). These frequencies and their associated severity ratings are summarized in **Figure 3**.

Within categories, aggressive behaviors were rated as moderate in 56% and severe in 30% of cases. Self-injurious behaviors were moderate in 54% and severe in 28%. Emotional dysregulation most frequently manifested as tantrums, while non-cooperative behaviors included struggles with transitions and elopement. A detailed breakdown of subtypes is available in **Table 2**.

**Table 2:** Behaviors of concern.

Child demographics	N = 498 (%)
Number of behaviors of concern	
1	103 (21)
2	93 (19)
3	86 (17)
4	68 (14)
5 or more	148 (30)
Number of Behaviors of Concern, median (IQR)	3 (1-4)
Overall behaviors of concern profile severity (highest severity level observed)^1^	
Mild	45 (9)
Moderate	261 (52)
Severe	192 (39)
Behaviors of concern categories	
Non-cooperative behaviors (NCB)	352 (71)
Emotional dysregulation behaviors (EDB)	348 (70)
Aggressive behaviors (AGB)	271 (54)
Self-injurious behaviors (SIB)	176 (35)
Inappropriate sexual behaviors (ISB)	31 (6)
Food-related behaviors (FRB)	22 (4)
Inappropriate toileting behaviors (ITB)	20 (4)
Behaviors of concern topologies	
Episode of emotional dysregulation (*e.g.*, tantrums; EDB)	330 (66)
Struggles with transitions (NCB)	243 (49)
Hitting others (AGB)	144 (29)
Bolts, wanders, elopes (NCB)	103 (21)
Destruction (EDB)	99 (20)
Extreme fixations (NCB)	94 (19)
Hitting head (SIB)	90 (18)
General aggression (AGB)	83 (17)
Refuses to engage (NCB)	53 (11)
Biting others (AGB)	43 (9)
Defying rules (NCB)	43 (9)
Kicks, trips, stomps others (AGB)	41 (8)
Hitting parts of the body (SIB)	36 (7)
Pushing others (AGB)	34 (7)
Self-biting (SIB)	31 (6)
Mean to others (AGB)	31 (6)
Pinching, digs nails into others (SIB)	29 (6)
Verbal abuse of others (AGB)	29 (6)
Scratching others (AGB)	22 (4)
Pulling others’ hair (AGB)	19 (4)
Skin picking (SIB)	17 (3)
Voiding, defecating not in the toilet (ITB)	16 (3)
Ingesting non-food items (FRB)	16 (3)
Self-scratching (SIB)	15 (3)
Grabbing others (AGB)	15 (3)
Self-pinching or pulling on the body (SIB)	13 (3)
Spitting (AGB)	13 (3)
Aggression toward animals (AGB)	11 (2)
Throws objects at others (AGB)	11 (2)
Inappropriate touching (ISB)	11 (2)
Hair pulling (SIB)	10 (2)
Suicidal behavior (SIB)	10 (2)
Suicidal ideation (SIB)	9 (2)
Inappropriate exposure (ISB)	9 (2)
Fecal smearing (ITB)	8 (2)
Negative self-talk (SIB)	8 (2)
Throwing body around (EDB)	8 (2)
Uncategorized self-injury (SIB)	8 (2)
Teeth grinding (SIB)	7 (1)
Threaten, gesturing to harm self (SIB)	7 (1)
Stealing (AGB)	7 (1)
Vomiting (FRB)	7 (1)
Uncategorized emotional dysregulation behaviors (EDB)	7 (1)
Uses objects as weapons (AGB)	≤5 (1)
Uncategorized sexual behaviors (ISB)	≤5 (1)
Inserting objects into body openings (SIB)	≤5 (1)
Pulling nails (SIB)	≤5 (1)
Touching genitals in public (ISB)	≤5 (1)
Lying (NCB)	≤5 (1)
Uncategorized inappropriate sexual behaviors (ISB)	≤5 (1)
Uncategorized oppositional behaviors (NCB)	≤5 (1)
Inserting fingers into body openings (SIB)	≤5 (1)
Extreme picky eating (FRB)	≤5 (1)
Headbutts (AGB)	≤5 (1)
Unwanted kissing (ISB)	≤5 (1)
Calling 911, pulling fire alarms (NCB)	≤5 (1)
Dangerous activity (EDB)	≤5 (1)
Twisting, bending fingers (SIB)	≤5 (1)
Charges at others (AGB)	≤5 (1)
Uncategorized other aggression (AGB)	≤5 (1)
Masturbation in public (ISB)	≤5 (1)
Pulling off others’ clothes (ISB)	≤5 (1)
Tasting or ingesting feces (ITB)	≤5 (1)
Rectal digging (ITB)	≤5 (1)

^1^Overall profile severity reflects the highest severity level of any documented behavior for the individual. Individuals with severe profiles may exhibit multiple mild or moderate behaviors but are classified as severe if at least one behavior meets severe criteria.

Missing data suppressed due to small (≤5) cell size.

AGB, aggressive behaviors; EDB, emotional dysregulation behaviors; FRB, food-related behaviors; ISB, inappropriate sexual behaviors; ITB, inappropriate toileting behaviors; NCB, non-cooperative behaviors; SIB, self-injurious behaviors.

### 3.5 Behavioral clustering

To examine the relationships among different categories of BoC, we calculated pairwise phi coefficients. The results indicated that certain behavior types frequently co-occurred, suggesting potential behavioral clustering. The strongest observed association was between aggression and emotional dysregulation (φ = 0.23), followed closely by aggression and self-injurious behaviors (φ = 0.20). Both values exceed conventional thresholds for clinical relevance and indicate moderate, meaningful associations between these behavior types.

**Figure 4:**
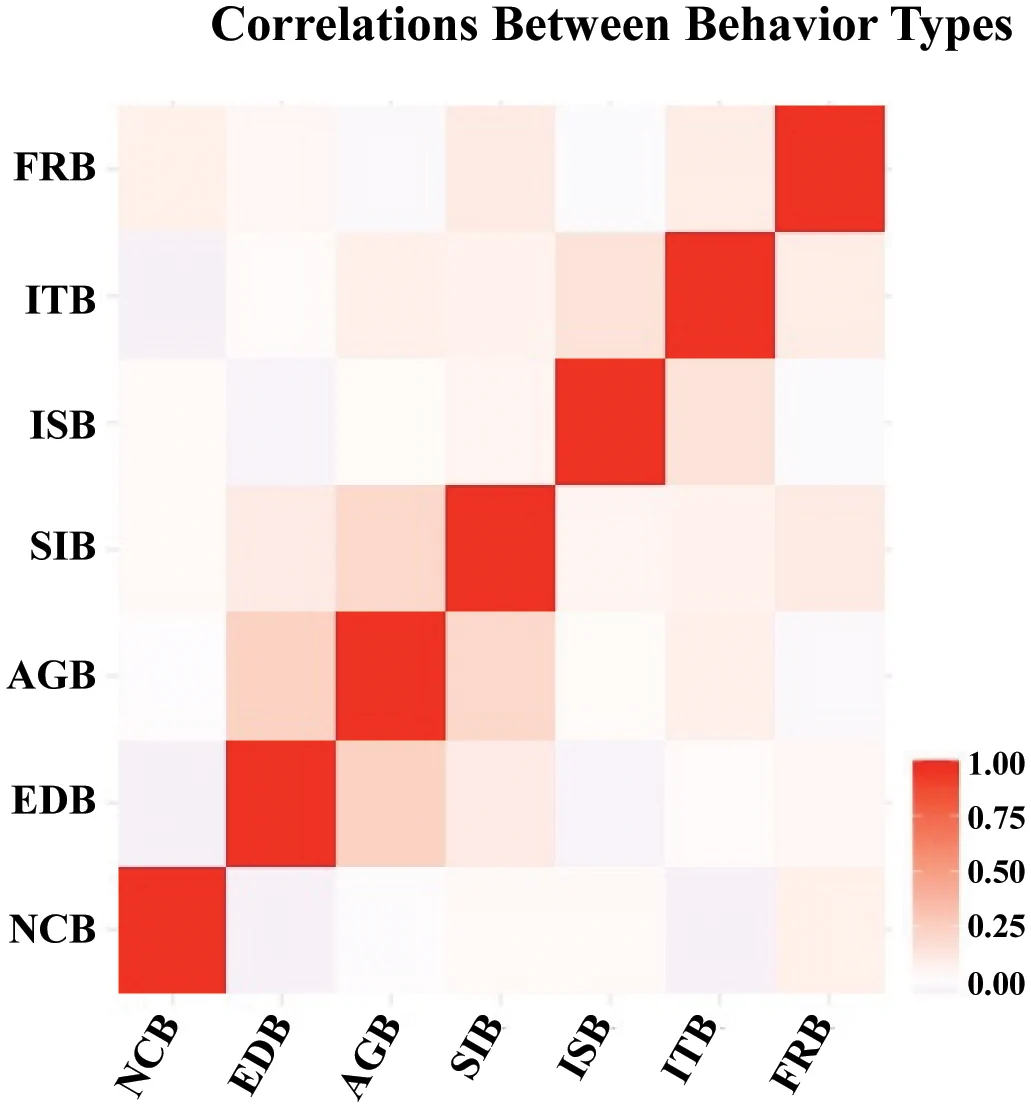
Correlations between behavior types. Phi coefficient matrix indicating the strength of association between different BoC types. Moderate associations were found between aggression and emotional dysregulation (φ = 0.23), and aggression and self-injurious behaviors (φ = 0.20), based on adequate sample sizes. Non-cooperative behaviors were not significantly associated with other behaviors. Associations involving low-frequency behaviors (*e.g.*, sexual, toileting, food) are considered unreliable. AGB, aggressive behaviors; EDB, emotional dysregulation behaviors; FRB, food-related behaviors; ISB, inappropriate sexual behaviors; ITB, inappropriate toileting behaviors; NCB, non-cooperative behaviors; SIB, self-injurious behaviors.

These findings suggest that children who exhibit aggressive behaviors are also likely to demonstrate emotional dysregulation and, to a lesser extent, self-injurious behaviors. In contrast, non-cooperative behaviors appeared to be largely independent of other BoC categories, showing minimal correlation across the matrix. Correlations involving less frequently reported behaviors, such as inappropriate sexual behaviors, toileting issues, and food-related concerns, were considered statistically unstable due to small sample sizes and should be interpreted with caution. The full correlation matrix is presented in **Figure 4** and **Supplementary Table 4**, with key associations described above.

### 3.6 Models of behavioral presentation

Across the four most prevalent BoC (emotional dysregulation, aggression, non-cooperation, and self-injury), the ML models achieved ROC-AUC values ranging from 0.60 to 0.69 for binary classification of each BoC presence. Several features consistently emerged as high-ranking model features (see **Figure 1**; **Table 3**). Sleep-related difficulties, particularly trouble falling asleep, appeared in all four models and were the most frequently shared features across all BoC. Speech and language delay were present in three of four models, ranking among the top features for both emotional dysregulation and self-injury. Autism-related traits, including restricted and repetitive behaviors, language impairment, and co-occurring NDDs, were common across BoC. In several models, both general autism diagnosis and specific DSM-5 criterion symptoms appeared as distinct features, indicating associations at multiple levels of diagnostic specificity. Engagement with rehabilitation and social services (*e.g.*, Pediatric Community Rehabilitation Services) was also common. GI problems were identified among high-ranking features for multiple behaviors, most notably aggression and self-injury. These associations suggest that somatic discomfort may contribute to externalizing and harm-related behaviors. These findings point to recurring patterns of sleep difficulties, communication impairments, somatic vulnerability, and high-intensity service involvement across diverse behavioral presentations.

**Table 3:** Machine learning model features for behaviors of concern.

Model features	Any BoC	Non-cooperative	Emotional dysregulation	Aggression	Self-injury	Sexual	Toileting	Food-related
Accuracy	65.224357	65.410163	62.415377	64.62623	59.210023	62.407413	74.12699	82.666667
Number of features	99.2	64.066667	77.7	76.166667	107.733333	83.633333	76.366667	19.433333
Model	nb	lmt	lmt	lmt	lmt	lmt	lmt	lmt
Feature selector	ReliefF	ReliefF	ReliefF	ReliefF	InfoGain	ReliefF	ReliefF	ReliefF
Minority class size^1^	208	512	512	544	346	54	42	40
Top 10 features	ADHD	Taking medication	Speech delay	Trouble falling asleep	Speech delay	Dermatologic	Autism B1 symptoms	Intellectual disability
	ADHD combined type	Autism specifier: co-occurrence	Night wakings	Autism B symptoms	Musculoskeletal problems	Autism B2 symptoms	Parent involvement	Specialized school
	Disability Tax Credit	Trouble falling asleep	Disability Tax Credit	Dermatologic	Respiratory problems	Night wakings	Autism B3 symptoms	Autism with Language impairment
	Hearing condition	Dysmorphisms	Total # supports	Pediatric community rehabilitation services	Language disorder	Family Support for Children with Disabilities	Seizures	Autism with ID
	Vision condition	Hem/Onc	Communication disorder	Intellectual disability	GI problems	Sleep medications	Speech language therapy	Intellectual disability
	Communication disorder	Communication disorder	Autism with Language impairment	HEENT problems	Autism B symptoms	GI problems	age	Trouble falling asleep
	GI problems	Speech delay	Pediatric community rehabilitation services	ADHD combined type	neurologic abnormal	Pediatric Community Rehabilitation Services	Autism B4 symptoms	Individualized Program Plan
	# of siblings	Speech language therapy	Trouble falling asleep	GI problems	Sleep medications	Trouble falling asleep	Specialized school	Night wakings
	Autism specifier: medical; genetic	Autism with language impairment	Autism B2 symptoms	Family Support for Children with Disabilities	Autism A symptoms	Genetic disorder	Psychologist	Disability Tax Credit
	Autism B3 symptoms	Hearing condition	Sleep problems	ADHD	Pediatric Community Rehabilitation Services	Sleep problems	Autism with language impairment	Autism

^1^Number reflects sample size per class after balanced sampling.

ADHD, attention-deficit/hyperactivity disorder; BoC, behaviors of concern; B1, deficits in social-emotional reciprocity; B2, deficits in nonverbal communicative behaviors; B3, deficits in developing, maintaining, and understanding relationships; B4, restricted, repetitive patterns of behavior, interests, or activities; GI, gastrointestinal; HEENT, head, eyes, ears, nose, and throat; Hem/Onc, hematology/oncology; ID, intellectual disability; InfoGain, Information Gain; lmt, linear model tree; nb, naive Bayes; ReliefF, ReliefF feature selection algorithm.

Each BoC ML model also made use of distinct feature profiles. For example, aggression was characterized by ADHD, intellectual disability, GI issues, and abnormal head and neck findings. Self-injury was associated with expressive language impairment and a broad set of physical exam abnormalities, including neurologic, musculoskeletal, respiratory, and GI problems. Emotional dysregulation included sleep maintenance difficulties and system-level complexity (*e.g.*, number of current supports) as high-ranking features. Non-cooperative behaviors were notably distinct, with top features including hearing impairments, dysmorphisms, hematological or oncologic conditions, and general medication use, features not identified in any other BoC ML model. Among the lower-frequency BoC categories used by the machine learning models, toileting concerns were associated with younger age, seizure disorders, and restricted and repetitive behaviors (B-symptoms) of autism (24); food-related behaviors were linked to autism and intellectual disability; and sexualized behaviors were uniquely predicted by genetic syndromes.

The composite ML model, which predicted the presence of any BoC, achieved a ROC-AUC of 0.66 and used ADHD, communication disorder, GI issues, and sensory impairments (hearing and vision) as high-ranking features. Other top-ranking features included autism-associated restricted interests, presence of a known medical or genetic contributor to autism, number of siblings, and receipt of the Disability Tax Credit (a federal credit that helps families of children with severe and prolonged impairments reduce their income tax burden). Of note, both ADHD (general diagnosis) and ADHD combined type appeared among the top features, a hierarchical pattern that indicates the model identified distinct information at both the broad diagnostic level and the specific subtype level. These features distinguished children with at least one BoC from those with no documented BoC and reflected a broadly distributed profile of neurodevelopmental, sensory, somatic, and family-level factors. Complete feature rankings and model performance metrics are presented in **Table 3**.

To examine broader clinical patterns across all BoC types, we grouped individual features into four clinically relevant domains based on conceptual similarity: Sleep Difficulties (sleep onset, maintenance, medications), Communication Impairment (expressive/receptive language, speech delays), Somatic Symptoms (pain, GI, sensory, motor problems), and System Complexity (service utilization, disability supports, care coordination). For each domain, we calculated two summary metrics: (1) average rank across all features within that domain when they appeared in top-10 features, and (2) total count of top-10 appearances across all BoC types. These domain-level aggregations (**Figure 1B**) complement the individual feature rankings (**Figure 1A**) by highlighting transdiagnostic patterns at a systems level.

## 4 Discussion

In this diagnostically complex, real-world cohort of children referred to tertiary developmental clinics, BoC were prevalent, often severe, and multifactorial in origin. Over 80% of children exhibited at least one BoC, most with moderate or severe intensity. Consistent with the younger age of this population, emotional dysregulation and non-cooperation were the most observed behaviors, followed by aggression and self-injury (3, 35). Nearly three-quarters of children presented with two or more NDDs, and co-occurring physical (80%) and mental health (21%) conditions were common. The burden of multiple behaviors was high (median 3 per child; 70% with more than one behavior, and over one-third with four or more). Moderate-to-severe intensity was predominant across all behavior types (>75%). This combination of multiplicity, severity, and diagnostic co-occurrence underscores the substantial clinical complexity facing providers. These findings highlight the need for transdiagnostic approaches to address overlapping behavioral concerns that move beyond diagnosis-specific frameworks (9, 20, 23, 36).

Neighborhood material and social deprivation, as measured by the MSDI, was also examined but did not emerge as a significant independent feature associated with the presence of BoC in this cross-sectional sample. However, this finding should be interpreted cautiously. Socioeconomic disadvantage exerts cumulative and delayed effects on developmental and behavioral trajectories, often manifesting beyond the early diagnostic period (37). Longitudinal follow-up is needed to fully assess the contribution of social determinants to behavioral complexity in neurodevelopmental populations.

Across all BoC, sleep disturbance emerged as a core transdiagnostic feature. Trouble falling asleep was the most important feature used by the ML models across BoC, while sleep medications and caregiver-reported night wakings were also used repeatedly. However, formal sleep disorder diagnoses were documented in only 12% of children despite 46% having caregiver-reported sleep disturbances, suggesting substantial underdiagnosis. This gap likely reflects multiple factors. First, behavioral sleep problems (difficulty initiating or maintaining sleep) are often managed empirically with behavioral strategies or pharmacotherapy without formal diagnostic coding. Second, children with neurodevelopmental disorders have elevated rates of obstructive sleep apnea, yet diagnostic polysomnography may be challenging due to limited pediatric sleep medicine access, cost barriers, and difficulty tolerating overnight monitoring, particularly relevant for children with sensory sensitivities, intellectual disability, or severe behavioral presentations (38, 39). Untreated sleep disorders, whether insomnia or sleep-disordered breathing, contribute significantly to daytime behavioral dysregulation and represent potentially modifiable targets for intervention. Our findings reinforce the need for systematic sleep screening in all children with BoC, particularly those with communication impairments or externalizing presentations.

Physical findings also featured prominently in our ML models. Abnormal GI, neurologic, and dermatologic examination findings were top features of self-injury and aggression, suggesting that possible somatic discomfort or sensory dysregulation may drive behavioral escalation, particularly in children with limited expressive communication capacity. GI problems (long documented in autism) ranked among the top features for multiple behavior types, while dermatologic conditions were uniquely associated with both aggression and sexualized behaviors. These findings may point to the importance of pain and interoceptive awareness in behavioral expression and the need to include physical health and sensory systems in routine behavioral assessments.

Communication impairments were another dominant feature set. Speech and language delays appeared among the highest-ranked features for emotional dysregulation and self-injury and were commonly used in most BoC models. While the association between communication impairments and BoC is well documented in autism (40), our findings suggest that communication difficulties may be equally associated with dysregulation in children across a range of NDDs, an area that has received comparatively less attention. Critically, these impairments are modifiable and should be assessed and treated proactively, rather than viewed as static correlates of underlying diagnoses (41).

This study included system-level features, such as engagement with pediatric rehabilitation services, family support programs, and disability tax benefits, which are relatively uncommon in research on BoC. These features were among the most consistent associations across models. They likely reflect not only functional impairment, but caregiver advocacy, chronicity of need, and difficulty navigating services (42, 43). Their associative value supports the inclusion of contextual and administrative data in clinical formulations and triage decisions.

Importantly, not all behaviors followed the same pattern. While aggression and emotional dysregulation shared common features and exhibited high behavioral co-occurrence (φ = 0.23), emotional dysregulation and non-cooperation, though associated with four overlapping top features, including communication and support variables, showed minimal behavioral co-occurrence. Despite overlapping features, emotional dysregulation and non-cooperation rarely co-occurred, highlighting that similar feature profiles can yield distinct behavioral expressions. Conversely, some behaviors revealed distinct, behavior-specific features. For example, non-cooperative behaviors were uniquely associated with hearing and vision impairments, dysmorphisms, and hematologic or oncologic diagnoses, associations not shared with any other BoC. These findings suggest that even within a transdiagnostic framework, some behaviors warrant targeted evaluation for underlying medical or sensory drivers.

Finally, the composite model predicting any BoC (*vs* none) included ADHD, communication disorder, autism-related restricted interests, GI/sensory impairments, and disability supports. This profile reinforces the multifactorial nature of behavioral vulnerability and identifies domains worth screening even before specific behaviors arise.

### 4.1 Limitations

This study has several limitations. First, the sample was drawn from children, mostly under age 10, referred for first-time developmental assessments at a single tertiary center. As such, these findings are not representative of the broader population of children with NDDs and should not be interpreted as prevalence estimates. Instead, the cohort reflects a high-need subgroup likely to require intensive services. Second, the study is subject to referral and selection bias, as children were referred based on clinician judgment and family concerns, which may vary by region, service availability, or provider. While this limits generalizability, it also reflects the reality of tertiary care practice and supports the goal of providing guidance relevant to high-need populations most likely to use intensive health, education, and social services. Third, behavior variables were derived from retrospective review of unstructured clinical records. Despite high inter-rater reliability, data quality was constrained by provider documentation practices, which varied in detail and comprehensiveness across clinicians and encounters. Nevertheless, these records represent the only information used to guide clinical decision-making in real-world practice. While prospective studies using standardized caregiver-report instruments would provide more systematic behavioral data, such measures are rarely implemented outside research settings, leaving substantial gaps in our understanding of diagnostically complex populations who do not meet the restrictive eligibility criteria typical of research cohorts. Finally, the use of supervised machine learning, while suitable for exploratory modeling in high-dimensional, observational data, does not support claims about direction or causality (33). These models were not externally validated, and findings should be interpreted as hypothesis-generating rather than definitive.

### 4.2 Conclusions

These findings support a shift from diagnosis-driven assessment and care toward a systems-informed model. Clinicians should screen for sleep disturbance, GI symptoms, pain/sensory reactivity, communication challenges, and systemic burden at intake. Early identification of modifiable contributors may improve outcomes for children with BoC, especially those seen in high-need, tertiary care settings.

Beyond these clinical implications, this study makes three broader contributions to the evidence base for neurodevelopmental populations. First, several well-established associations with behavioral severity from diagnostically homogeneous research samples (*e.g.*, sleep difficulties, GI problems, communication impairments) replicated in this heterogeneous clinical population. This convergence suggests that findings from more restrictive research cohorts may generalize to the complex, co-occurring presentations encountered in real-world practice. Second, clinical documentation, while imperfect and subject to documentation bias, can be systematically abstracted to identify actionable patterns that inform resource allocation and care planning. This is particularly relevant given that standardized behavioral assessment instruments are rarely implemented outside research settings, leaving substantial gaps in our understanding of diagnostically complex populations. Third, the transdiagnostic nature of these associations, appearing consistently across children with diverse NDD profiles and co-occurring conditions, indicates that behavioral associations may be more productively understood and addressed at a systems level rather than within diagnosis-specific frameworks.

These findings are necessarily exploratory and hypothesis-generating given the retrospective, cross-sectional design and reliance on clinical records. However, they demonstrate that clinically meaningful signals can be extracted from real-world populations served in tertiary care: notably, populations that have been systematically excluded from much of the existing evidence base. Future research should prospectively validate these associations using standardized caregiver-report measures and examine whether early intervention targeting modifiable factors reduces BoC severity and improves functional outcomes over time.

## Data Availability

The datasets presented in this article are not readily available because of patient confidentiality, participant privacy, limitations of Alberta’s Health Information Act, and the study’s ethics approval. Requests to access the datasets should be directed to sarah.maceachern@ucalgary.ca.
