# Supplementary material for: Transdiagnostic clinical correlates for behaviors of concern in pediatric neurodevelopmental disorders: a retrospective chart review and machine learning analysis
Source: Front Psychiatry. 2026 Aug 12;17:1739597. doi: 10.3389/fpsyt.2026.1739597 (PMC13506917; doi:10.3389/fpsyt.2026.1739597)
Supplement: Supplementary file 1 [file DataSheet1.docx]

Supplementary Material

# 1 Supplementary Data

## 1.1 Study Design and Setting

We conducted a retrospective chart review of children aged 2 to 17 years who underwent first-time diagnostic assessments in subspecialty developmental pediatrics clinics at Alberta Children’s Hospital between May 31, 2022, and May 30, 2023. Eligible patients were required to have:

•	A documented neurodevelopmental disorder (NDD) diagnosis.

•	Participation in one of the following consultative clinics:

•	Developmental Neurology Clinic

•	Pediatric Oncology Neurodevelopmental Clinic

•	Complex Medical and Genetic Clinic

•	Cumulative Risk Diagnostic Clinic

•	Developmental Pediatric Psychopharmacology Clinic

•	Neurodevelopmental Attention Clinic

•	Autism Spectrum Diagnostic Clinic

These clinics collectively provide comprehensive diagnostic services to children with a range of developmental, medical, genetic, and behavioral complexities. Patients referred to these clinics are typically seen for interdisciplinary evaluations. Only first-time assessments were included to standardize baseline diagnostic profiles and minimize confounding from prior specialist involvement.

## 1.2 Demographics

Neighborhood socioeconomic status was assessed using the Material and Social Deprivation Index (MSDI), a validated composite index incorporating indicators of material and social disadvantage (1, 2). Data were obtained from the 2021 Canadian census and matched to study participants via postal codes. The MSDI is based on dissemination areas (DAs), the smallest geographical units provided by Statistics Canada, ensuring socioeconomic homogeneity within each unit.

The MSDI comprises six standardized indicators: 1) proportion of individuals aged 15 years and older without a high school diploma, 2) employment-to-population ratio for individuals aged 15 years and older, 3) average income for the population aged 15 years and older, 4) proportion of individuals aged 15 years and older living alone, 5) proportion of individuals aged 15 years and older who are separated, divorced, or widowed, and 6) proportion of single-parent families.

Following the recommended methodology (2), these indicators were combined into two dimensions (material and social deprivation) using Principal Component Analysis (PCA). Each dissemination area was ranked according to its factor score on both dimensions, then grouped into quintiles, with quintile 1 representing the most privileged areas and quintile 5 the most deprived. A combined deprivation index was then constructed by cross-tabulating the quintiles of material and social deprivation, resulting in a matrix identifying varying levels of overall deprivation.

Of note, postal codes for 24 participants could not be matched to existing dissemination areas, as these represented newly established postal codes not yet incorporated into the 2021 census database. These participants were therefore excluded from the MSDI analysis due to missing data, and this exclusion is noted in the descriptive statistics.

# 2 References

1.	Pampalon R, Hamel D, Gamache P, Philibert MD, Raymond G, Simpson A. An area-based material and social deprivation index for public health in Quebec and Canada. Can J Public Health. 2012;103(8 Suppl 2):S17-22.

2.	Gamache P, Hamel, D., Blaser, C. Material and social deprivation index: A summary –INSPQ Website. 2019.

# 3 Tables

**Supplementary Table 1: Most Common Pairs of Co-occurring NDD Diagnoses**

| **NDD Diagnostic Combinations in Children with ≥2 Diagnoses** | | |
| --- | --- | --- |
| **Diagnosis 1** | **Diagnosis 2** | **N=436 (%)** |
| Autism | Communication disorder | 178 (41) |
| Autism | Intellectual disability | 166 (38) |
| Autism | ADHD | 96 (22) |
| Intellectual disability | Communication disorder | 95 (22) |
| ADHD | Communication disorder | 69 (16) |
| Intellectual disability | ADHD | 62 (14) |
| Specific learning disorder | ADHD | 43 (10) |
| ADHD | Motor disorder | 36 (8) |
| Communication disorder | Motor disorder | 30 (7) |
| Autism | Motor disorder | 27 (6) |
| Intellectual disability | Motor disorder | 19 (4) |
| Autism | Specific learning disorder | 15 (4) |
| Specific learning disorder | Communication disorder | 15 (3) |
| Specific learning disorder | Motor disorder | 13 (3) |
| ADHD | ND-PAE | 13 (3) |
| ND-PAE | Communication disorder | 9 (2) |
| Intellectual disability | Specific learning disorder | 8 (2) |
| Intellectual disability | ND-PAE | 7 (2) |
| ND-PAE | Motor disorder | 7 (2) |
| Specific learning disorder | ND-PAE | 6 (1) |
| Cerebral Palsy | Intellectual disability | ≤5 (1) |
| Autism | ND-PAE | ≤5 (1) |
| Cerebral Palsy | Communication disorder | ≤5 (1) |

Missing data suppressed due to small (≤5) cell size.

**Abbreviations:** ADHD, attention-deficit/hyperactivity disorder; ND-PAE, neurodevelopmental disorder associated with prenatal alcohol exposure; NDD, neurodevelopmental disorder.

**Supplementary Table 2: Most Common Triplets of Co-occurring NDD Diagnoses**

| **NDD Diagnostic Combinations in Children with ≥3 Diagnoses** | | | |
| --- | --- | --- | --- |
| **Diagnosis 1** | **Diagnosis 2** | **Diagnosis 3** | **N=162 (%)** |
| Autism | Communication disorder | Intellectual disability | 63 (39) |
| Autism | ADHD | Communication disorder | 33 (20) |
| Autism | ADHD | Intellectual disability | 32 (20) |
| ADHD | Communication disorder | Intellectual disability | 24 (15) |
| Autism | Communication disorder | Motor disorder | 15 (9) |
| ADHD | Communication disorder | Specific learning disorder | 15 (9) |
| Autism | ADHD | Specific learning disorder | 13 (8) |
| ADHD | Motor disorder | Specific learning disorder | 13 (8) |
| ADHD | Communication disorder | Motor disorder | 13 (8) |
| ADHD | Intellectual disability | Motor disorder | 12 (7) |
| Autism | Intellectual disability | Motor disorder | 11 (7) |
| Autism | ADHD | Motor disorder | 11 (7) |
| Communication disorder | Intellectual disability | Motor disorder | 11 (7) |
| ADHD | Communication disorder | ND-PAE | 7 (4) |
| ADHD | Intellectual disability | Specific learning disorder | 6 (4) |
| ADHD | Intellectual disability | ND-PAE | 6 (4) |
| ADHD | ND-PAE | Specific learning disorder | 6 (4) |
| ADHD | Motor disorder | ND-PAE | 6 (4) |
| Communication disorder | Intellectual disability | ND-PAE | ≤5 (1) |
| Communication disorder | Motor disorder | Specific learning disorder | ≤5 (1) |
| Communication disorder | Motor disorder | ND-PAE | ≤5 (1) |

Missing data suppressed due to small (≤5) cell size.

**Abbreviations:** ADHD, attention-deficit/hyperactivity disorder; ND-PAE, neurodevelopmental disorder associated with prenatal alcohol exposure; NDD, neurodevelopmental disorder.

**Supplementary Table 3: Most Common Pairs of Co-occurring Physical Health Conditions**

| **Physical Health Combinations in Children with ≥2 Conditions** | | |
| --- | --- | --- |
| **Diagnosis 1** | **Diagnosis 2** | **N=324 (%)** |
| Sleep problem | GI problem | 125 (39) |
| Vision condition | Sleep problem | 76 (23) |
| Sleep disorder | Sleep problem | 66 (20) |
| Allergies | Sleep problem | 53 (16) |
| Vision condition | GI problem | 52 (16) |
| Genetic disorder | Sleep problem | 47 (14) |
| Sleep disorder | GI problem | 40 (12) |
| Genetic disorder | GI problem | 38 (12) |
| Allergies | GI problem | 34 (10) |
| Genetic disorder | Vision condition | 29 (9) |
| Asthma | Sleep problem | 27 (8) |
| Dermatologic condition | Sleep problem | 24 (7) |
| Sleep problem | Dental | 24 (7) |
| Seizures | Sleep problem | 23 (7) |
| Hearing condition | Sleep problem | 23 (7) |
| Asthma | GI problem | 22 (7) |
| Seizures | GI problem | 22 (7) |
| Genetic disorder | Sleep disorder | 21 (6) |
| Vision condition | Sleep disorder | 21 (6) |
| Sleep problem | Hematological/Oncology | 21 (6) |
| Hearing condition | Vision condition | 18 (6) |
| Allergies | Vision condition | 18 (6) |
| Genetic disorder | Allergies | 17 (5) |
| Dermatologic condition | GI problem | 17 (5) |
| GI problem | Dental | 16 (5) |
| Seizures | Vision condition | 16 (5) |
| Allergies | Sleep disorder | 16 (5) |
| Headache disorders | Sleep problem | 16 (5) |
| Genetic disorder | Hearing condition | 15 (5) |
| Asthma | Allergies | 15 (5) |
| GI problem | Hematological/Oncology | 15 (5) |
| Hearing condition | GI problem | 14 (4) |
| Cardiovascular | Sleep problem | 14 (4) |
| Headache disorders | GI problem | 14 (4) |
| Allergies | Dermatologic condition | 13 (4) |
| Cardiovascular | Vision condition | 13 (4) |
| Genetic disorder | Asthma | 12 (4) |
| Genetic disorder | Seizures | 12 (4) |
| Genetic disorder | Cardiovascular | 12 (4) |
| Asthma | Vision condition | 12 (4) |
| Sleep problem | Musculoskeletal condition | 12 (4) |

**Abbreviations:** GI, gastrointestinal

**Supplemental Table 4: Phi Correlation Matrix**

|  | **NCB** | **EDB** | **AGB** | **SIB** | **ISB** | **ITB** | **FRB** |
| --- | --- | --- | --- | --- | --- | --- | --- |
| **NCB** | 1 | -0.05 | -0.01 | 0.02 | 0.02 | -0.05 | 0.08 |
| **EDB** | -0.05 | 1 | 0.23 | 0.1 | -0.05 | 0.01 | 0.03 |
| **AGB** | -0.01 | 0.23 | 1 | 0.2 | 0.02 | 0.08 | -0.02 |
| **SIB** | 0.02 | 0.1 | 0.2 | 1 | 0.05 | 0.07 | 0.11 |
| **ISB** | 0.02 | -0.05 | 0.02 | 0.05 | 1 | 0.15 | -0.01 |
| **ITB** | -0.05 | 0.01 | 0.08 | 0.07 | 0.15 | 1 | 0.1 |
| **FRB** | 0.08 | 0.03 | -0.02 | 0.11 | -0.01 | 0.1 | 1 |

**Abbreviations:** AGB, aggressive behaviors; EDB, emotional dysregulation behaviors; FRB, food-related behaviors; ISB, inappropriate sexual behaviors; ITB, inappropriate toileting behaviors; NCB, non-cooperative behaviors; SIB, self-injurious behaviors.
